# Intensive surveillance endoscopy for multiple gastrointestinal tumors in a patient with constitutional mismatch repair deficiency: case report

**DOI:** 10.1186/s12876-021-01902-6

**Published:** 2021-08-23

**Authors:** Takayuki Ando, Takahiko Nakajima, Rei Fukuda, Keiko Nomura, Yo Niida, Miho Sakumura, Iori Motoo, Hiroshi Mihara, Sohachi Nanjo, Shinya Kajiura, Haruka Fujinami, Shojo Hojo, Tsutomu Fujii, Ichiro Yasuda

**Affiliations:** 1 Third Department of Internal Medicine, University of Toyama, 2630 Sugitani, 930-0194 Toyama, Japan; 2 Department of Diagnostic Pathology, Graduate School of Medicine and Pharmaceutical Sciences, University of Toyama, Toyama, Japan; 3 Department of Clinical Genetics, Toyama University Hospital, Toyama, Japan; 4 Department of Pediatrics, University of Toyama, Toyama, Toyama Japan; 5 Division of Genomic Medicine, Department of Advanced Medicine, Medical Research Institute, Kanazawa Medical University, Uchinada, Ishikawa Japan; 6 Department of Surgery and Science, Faculty of Medicine, Academic Assembly, University of Toyama, Toyama, Japan

**Keywords:** Colorectal polyposis, Constitutional mismatch repair deficiency, Surveillance endoscopy, Lynch syndrome, Case report

## Abstract

**Background:**

Constitutional mismatch repair deficiency (CMMRD) is an extremely rare autosomal recessive hereditary disease characterized by the absence of mismatch repair gene activity from birth, which results in brain tumors, colonic polyposis, gastrointestinal cancers, and lymphomas later in life. An aggressive approach, including colectomy or proctocolectomy, is recommended for the treatment of colorectal cancer. Additionally, partial colectomy with subsequent endoscopic surveillance may be an alternative strategy due to poor patient’s condition, although there is no evidence of surveillance endoscopy after partial colectomy for CMMRD.

**Case presentation:**

A 13-year-old male patient with a history of T-lymphoblastic lymphoma underwent total gastrointestinal endoscopy, which revealed rectal cancer, colorectal polyposis, and duodenal adenoma. Differential diagnosis included constitutional mismatch repair deficiency according to its scoring system and microsatellite instability, and subsequent germline mutation testing for mismatch repair genes confirmed the diagnosis of constitutional mismatch repair deficiency based on a homozygous mutation in mutS homolog 6 (*MSH6*). The patient and his family refused colectomy due to the high risk of malignancies other than colorectal cancer, which could require radical surgery. Therefore, the patient underwent low anterior resection of the rectosigmoid colon for rectal cancer and intensive surveillance endoscopy for the remaining colon polyposis. During the 3-year period after initial surgery, 130 polyps were removed and the number of polyps gradually decreased during 6-months interval surveillance endoscopies, although only one polyp was diagnosed as invasive adenocarcinoma (pT1).

**Conclusions:**

Our experience of short surveillance endoscopy illustrates that this strategy might be one of options according to patient’s condition.

## Background

Lynch syndrome is a cancer predisposition syndrome caused by heterozygous germline mutations in DNA mismatch repair (MMR) genes, including mutL homolog 1 (*MLH1*), mutS homolog 2 (*MSH2*), *MSH6*, PMS1 homolog 2 (*PMS2*), and epithelial cell adhesion molecule (*EPCAM*) [1–3]. Conversely, constitutional mismatch repair deficiency (CMMRD) is an extremely rare autosomal recessive hereditary disease. Individuals with biallelic MMR mutations develop gastrointestinal polyposis; early-onset brain, hematological, and gastrointestinal cancers; and neurofibromatosis 1-like phenotype including café au lait macules [4–6]. In a cohort of 24 individuals with CMMRD, the International Hereditary Biallelic Mismatch Repair Deficiency (BMMRD) Consortium reported that almost 80% of patients with CMMRD had gastrointestinal adenomas or cancers and that 60% of patients had non-gastrointestinal cancers including lymphoma, leukemia, and brain cancer [7]. Therefore, treatment strategies for colorectal cancer include careful evaluation of the entire gastrointestinal tract and consideration of the patient’s general condition and risk of other neoplasms, although colectomy or proctocolectomy is recommended^4^. If neither procedure can be performed due to the patient’s condition, partial colectomy with subsequent endoscopic surveillance may be an alternative strategy [4]. However, the clinical course after partial colectomy remains unclear. Herein, we report the 3-year intensive endoscopic surveillance results after partial colectomy for early rectal cancer in a patient with CMMRD-associated multiple gastrointestinal tumors.

## Case presentation

A 6-year-old boy was diagnosed with T-lymphoblastic lymphoma and received chemotherapy and radiotherapy; he achieved complete remission and underwent annual follow-up with positron emission tomography/computed tomography (CT) using 18-fluoro-2-deoxyglucose. At the age of 13, he was referred to our department for further evaluation of a rectal tumor identified by positron emission tomography/CT.

**Fig. 1:**
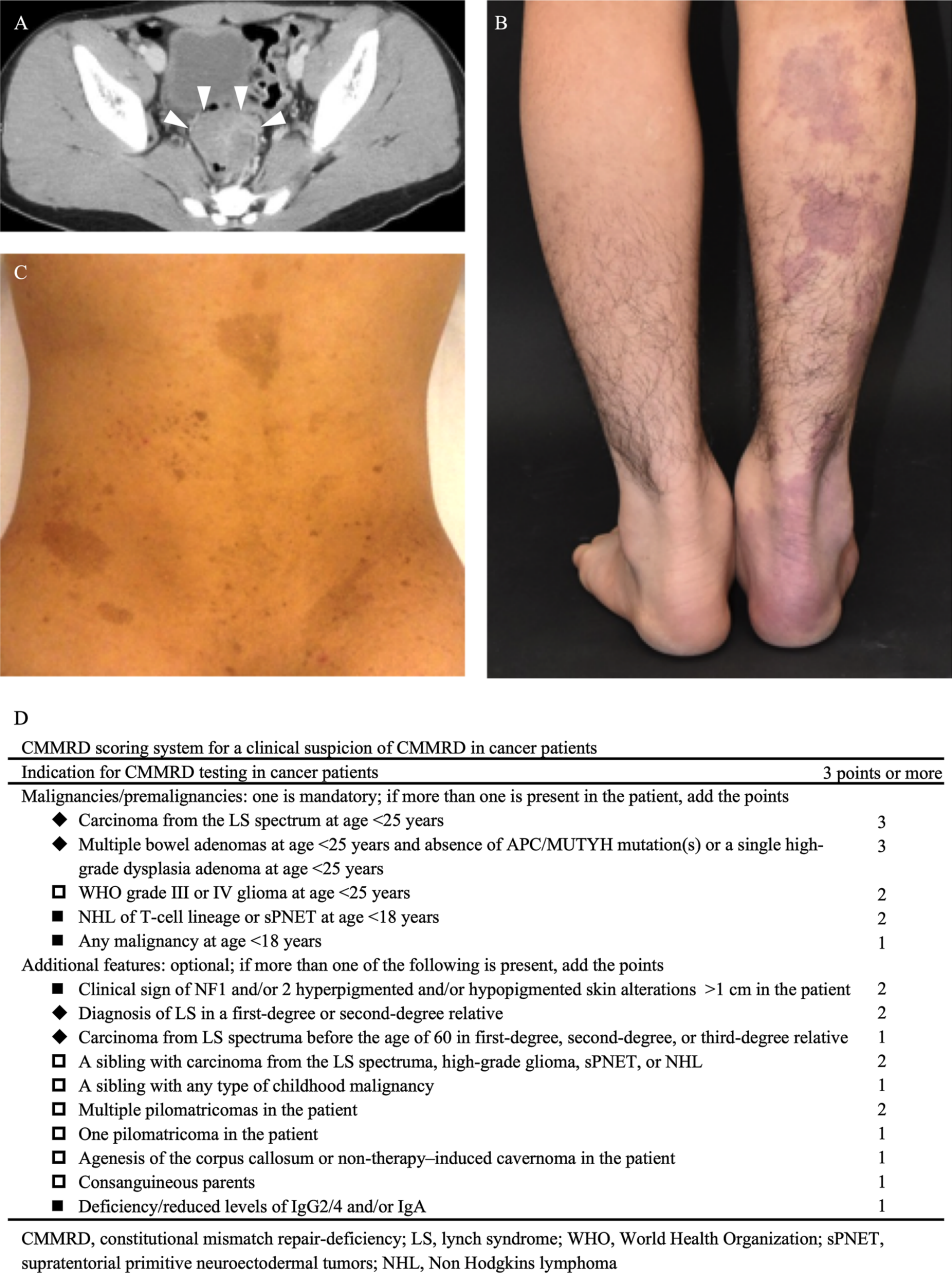
Findings of computed tomography scan, physical examination, and CMMRD scoring system. **A** Abdominal computed tomography image showing rectal wall thickening without lymphadenopathy (white arrowheads). **B**, **C** On physical examination, several café au lait macules and multiple cutaneous hemangiomas on back and right leg, respectively, are visible. **D** The patient had scores of 5 and 14 points, before and after genetic testing, respectively, according to the CMMRD scoring system. ■, criteria fulfilled before genetic testing; ◆, criteria fulfilled after genetic testing; □, unfulfilled criteria; CMMRD, constitutional mismatch repair deficiency

**Fig. 2:**
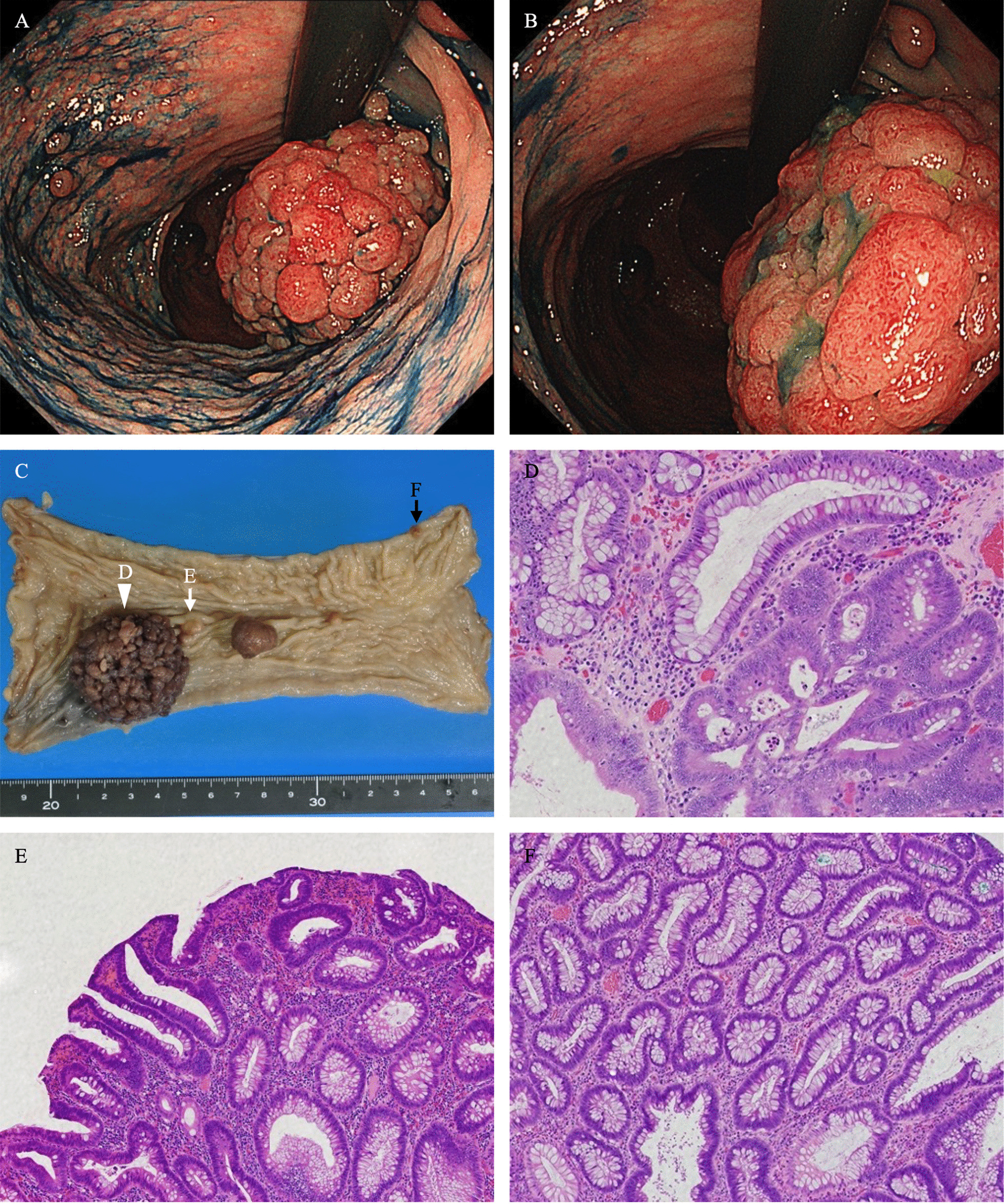
Colonoscopic and pathological findings of colorectal polyps. **A**, **B** Colonoscopy showing a sessile polyp with a central depression in the rectosigmoid area and multiple polyps. **C** Gross image of the resected rectosigmoid colon. A total of 13 polyps, with maximum diameters of up to 45 mm, are found. **D** Microscopic view of the largest polyp (white arrowhead in **B**). Intramucosal adenocarcinoma (pT1) with complex cribriform architecture in adenoma. **E**, **F** Microscopic view of other polyps (black and white arrows in **C**). Low-grade tubular adenoma

Physical examination revealed several café au lait macules and multiple cutaneous hemangiomas. Abdominal enhanced CT revealed irregular rectal wall thickening without lymphadenopathy or distant metastases (Fig. 1A–C). Total colonoscopy and endoscopic biopsy revealed a 45-mm sessile adenocarcinoma with a central depression in the rectosigmoid area and more than 100 adenomatous polyps, 2–15 mm in size, distributed throughout the colon and rectum (Fig. 2A, B). Additionally, one duodenal adenoma, 10 mm in size, was identified by esophagoduodenoscopy and capsule endoscopy. Serum levels of immunoglobulin G and G4 were 434 (normal, 870–1700) and < 2.0 (normal, 11–121) mg/dL, respectively.

**Fig. 3:**
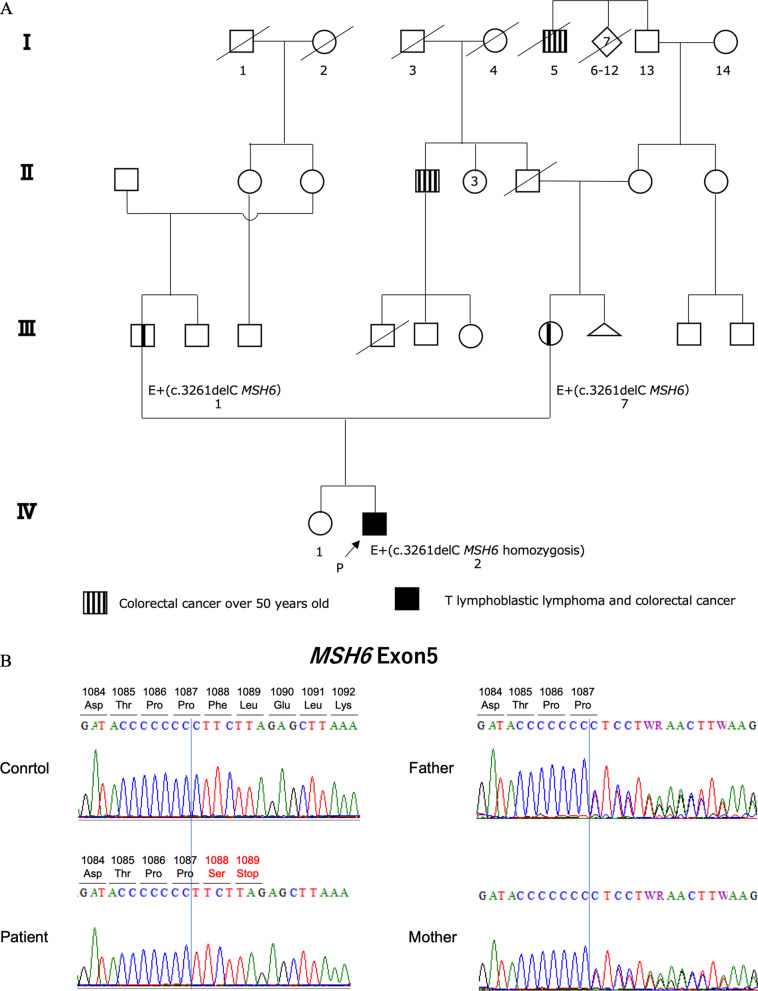
Family pedigree and genetic testing. **A** Proband’s pedigree revealed colon cancer findings in their seventies and eighties, respectively. **B** Sanger sequencing confirmed a homozygous mutation in *MSH6* exon 5. The deletion of C at 3261 results in an amino acid change from CCC to CCT and a change in amino acid 1089 to the stop codon (TAG). The parents of the patient are carriers of the *MSH6 c.*3261delC mutation, *MSH6*, mutS homolog 6

CMMRD was considered in the differential diagnosis based on these findings and the CMMRD scoring system, although the patient did not fulfill the Amsterdam criteria for Lynch syndrome (Fig. 1D) [8, 9]. Therefore, testing after obtaining informed consent revealed the rectal cancer harbored high microsatellite instability. After obtaining further written informed consent, subsequent genetic testing performed for hereditary colorectal cancer syndromes, including those associated with mismatch repair genes (*MLH1, MSH2, PMS2*, and *MSH6*), adenomatous polyposis coli, and mutYH-associated polyposis revealed one homozygous frame shift mutation in *MSH6* (NM_000179.2; c.3261del p.Phe1088SerfsTer2), which was reported as pathogenic (ClinVar accession VCV000089363.14, https://www.ncbi.nlm.nih.gov/clinvar/, last accessed May 15, 2021), confirming the diagnosis of CMMRD (Fig. 2 A). Genetic testing showed that the patient’s mother and father were heterozygous carriers of the *MSH6* c.3261del mutation, leading to the diagnosis of Lynch syndrome based on the same germline mutation in both parents (Fig. 3A, B). Both parents had normal findings by colonoscopy and esophagoduodenoscopy.

The patient and his parents were carefully and repeatedly informed about the risks and benefits of colectomy and proctocolectomy for rectal cancer and colorectal polyposis, which they declined because of the high risk of small intestinal and non-gastrointestinal cancers in future. Therefore, the patient underwent low anterior resection (LAR) of the rectosigmoid colon for rectal cancer and surveillance endoscopy was planned for the remaining colon polyposis and duodenal adenoma. Histopathologically, the excised rectal mass was an intramucosal, moderately differentiated tubular adenocarcinoma (pTis) in tubulovillous adenoma without lymph node metastasis. The patient was diagnosed with Stage I rectal cancer according to the Tumor-Node-Metastasis staging system (Fig. 2C–F). After surgery, colonoscopy was planned with the following goals: (1) polypectomy every 4–6 months until only polyps sized < 4 mm remained; (2) histological evaluation of polyps sized > 10 mm and polyps sized < 10 mm with suspicion of cancer based on endoscopic appearance [10].

During the first colonoscopy after surgery, two polyps were pathologically diagnosed as intramucosal adenocarcinoma (pTis) in adenoma. However, in the third colonoscopy, one polyp, sized 8 mm, in descending colon was diagnosed as invasive adenocarcinoma without lymphovascular invasion (> 1000 μm, pT1). The patient refused the recommended additional surgery including colectomy. Subsequently, surveillance endoscopy and CT examination were continued, and lymph node and distant metastases were not present at last follow-up at three years after initial surgery. During the 3-year follow-up period with short-interval surveillance colonoscopy, 130 colon polyps were removed without adverse events, and the number of detected polyps gradually decreased (Table 1). The duodenal adenoma was treated with endoscopic mucosal resection after LAR. Until the last follow-up, the subsequently performed annual esophagoduodenoscopy and capsule endoscopy led to the identification of three duodenal adenomas, sized < 5 mm, which were removed.

**Table 1:** Clinicopathological characteristics of colon polyps treated during surveillance colonoscopy after surgery

Session	Months after surgery	Total number of colon polyps removed	Carcinoma diagnosis	Carcinoma characteristics					
				Location	Growth type	Size (mm)	pT stage*	Horizontal margin	Vertical margin
1	2	22	Yes	S	Polypoid	20	pTis	(−)	(−)
				R	Polypoid	17	pTis	(−)	(−)
2	7	42	No						
3	12	23	Yes	D	LST-NG	8	pT1	(ND)	(ND)
4	18	12	No						
5	24	12	No						
6	30	19	No						

*S* sigmoid colon, *R* rectum, *D* descending colon, *LST-NG* laterally spreading tumor non-granular type, *ND* not determined

*Based on classification system by the Union for International Cancer Control

## Discussion and conclusions

Recent studies suggest that > 50%, 40%, and 30% of patients with CMMRD develop malignant brain tumors, gastrointestinal tumors, and hematological malignancies, all during childhood, reflecting the generally poor prognosis of CMMRD [4]. The most frequent CMMRD-associated cancers are brain glioma diagnosed at 9.5 years of age, non-Hodgkin’s lymphoma diagnosed at 5 years of age, and colorectal cancer diagnosed at 16 years of age [11]. Besides very high tumor risks, CMMRD phenotypes are often characterized by the presence of signs reminiscent of neurofibromatosis type 1 [8]. The present 13-year-old patient with CMMRD and history of T-lymphoblastic lymphoma is a case of colon polyposis caused by biallelic germline mutation in an MMR gene.

The management of colon cancer in patients with CMMRD is based on the frequency of synchronous or metachronous gastrointestinal and non-gastrointestinal cancers. The International BMMRD Consortium reported that the approximate frequencies of synchronous and metachronous colorectal cancers were 20 and 50%, respectively [7]. Therefore, an aggressive approach, including colectomy with ileorectal anastomosis or proctocolectomy and construction of an ileal pouch-anal anastomosis, is recommended for colon polyposis in CMMRD patients [5]. Moreover, close monitoring of the rectum with endoscopy every 6 or 12 months is crucial after ileorectal anastomosis. However, metachronous non-colorectal cancers are frequent in patients with CMMRD [12]. Among eight patients with CMMRD and colorectal cancer, small intestinal and non-gastrointestinal cancers were diagnosed after the treatment of colorectal cancer in one and three patients, respectively [7]. Moreover, the cancer spectrum is reported to be related to specific MMR gene mutations. *MSH6* and/or *PMS2* mutations lead to cancers within ten years of life, and 34% of patients with *MSH6* mutations develop a second metachronous malignancy. The current patient and his parents refused colectomy after considering the high risk of metachronous cancers including non-gastrointestinal cancers.

In the present patient, the treatment strategy for colon polyposis was based on the endoscopic treatment for familial adenomatous polyposis (FAP), in which colectomy is a standard approach to prevent colorectal cancer [13]. However, colectomy is also associated with morbidity and mortality and removal of the large intestine affects quality of life [14, 15]. Therefore, the efficacy and safety of endoscopic management for colon clearance was considered in the current patient. In a study of patients with FAP refusing surgery, invasive colorectal cancer was not observed during a median follow-up of 5.1 years and there were no complications, suggesting that endoscopic management might prevent cancer development in patients with FAP [10, 16]. On the other hand, partial colectomy with subsequent regular surveillance colonoscopy is recommended in patients with Lynch syndrome and colorectal cancer, although the appropriate interval of surveillance colonoscopy after partial colectomy remains unclear [17]. However, colonoscopy performed in 6-month intervals was occasionally insufficient to detect endoscopically resectable tumors in some patients with high risk Lynch syndrome. Indeed, the present patient was diagnosed with an invasive cancer in descending colon during third colonoscopy after LAR. Additional surgery should be done in cases of endoscopically resected T1 cancer with positive vertical margin, although the relapse ratio of approximately 3.4% is relatively low [18]. Therefore, our strategy should be considered when colectomy is not appropriate due to patient’s condition.

Recent studies analyzing the association between tumor genetics and clinical spectrum should lead to the development of appropriate treatment strategies in patients with CMMRD [19]. In a study utilizing next-generation sequencing of 17 high-grade brain tumors in patients with CMMRD, the tumors exhibited massive numbers of substitution mutations (average, 7911 coding mutations; 249 mutations/Mb), which were higher than that observed in tumors of patients without CMMRD (0.61 mutations/Mb); the CMMRD-associated tumors were termed ultra-hypermutated cancers [20]. Moreover, these cancers acquired driver mutations in DNA polymerase ɛ (*POLE*) or δ (*POLD1*), which appeared to result in the loss of replication fidelity and a high mutation rate [21]. Gastrointestinal polyps without *POLE* and *POLD1* mutations in patients with CMMRD did not exhibit higher mutational loads. Another study reported differences in the prevalence rates of hematological, brain, and Lynch syndrome-associated cancers among patients with CMMRD harboring *MLH1*/*MSH2, MSH6*, and *PMS2* mutations^6^. These results should contribute to the adjustment of treatment modalities, offering surveillance strategies for second malignancies and appropriate counseling of the entire family.

In the present patient with CMMRD and colon polyposis, intensive surveillance endoscopy for multiple gastrointestinal tumors enabled the reduction in the number of lesions. The standard of care should be colectomy or protocolectomy for colorectal polyposis in patients with CMMRD. However, our experience of short surveillance endoscopy illustrates that our strategy might be one of options according to patient’s condition.

## Data Availability

The datasets used and/or analyzed during this study are included in this paper and shall be available from the corresponding author upon request.

## Abbreviations

CMMRD: Constitutional mismatch repair deficiency
CT: Computed tomography
LAR: Low anterior resection
FAP: Familial adenomatous polyposis
